# Nursing students’ willingness to respond in disasters: a cross sectional study of facilitators and barriers

**DOI:** 10.1186/s12912-024-02088-4

**Published:** 2024-06-20

**Authors:** Zahra Tayebi, Roohangiz Norouzinia, Zahra Moatadelro, Ashkan Farokhi Pour, Bahar Nourian

**Affiliations:** 1 School of Nursing and Midwifery, Alborz university of Medical Sciences, Karaj, IR Iran; 2 Social Determinants of Health Research Center, Alborz university of Medical Sciences, Saffarian St. 45 Metri Golshahr, Karaj, IR Iran

**Keywords:** Respond, Disasters, Nursing students, Willingness, Participation

## Abstract

**Introduction:**

The preparedness of the healthcare system to address emergency situations is contingent on the inclination of healthcare personnel. Nursing students can serve as valuable resources to supplement the workforce during major incidents and disasters. This study investigated the facilitators and barriers of nursing students’ willingness to respond to disasters at the Alborz University of Medical Sciences in 2022.

**Methods:**

In this cross-sectional descriptive study, 234 nursing students were recruited using convenience sampling. A deductive-inductive questionnaire was developed and distributed through an online self-administered survey comprising demographic information and questions on barriers, facilitators, various disaster scenarios, preferred activities, and reasons for pursuing a nursing career.

**Results:**

The mean willingness scores of nursing students in response to various disasters were as follows: 3.15 for natural disasters, 2.60 for man-made disasters, 2.94 for pandemics, and 3.32 overall. Among the disaster scenarios, the earthquake response obtained the highest willingness score, while infectious disease epidemics received the lowest score. The most and least willingness to perform activities during disaster response were related to bedside care and participation in patients’ personal hygiene, respectively. Key determinants of participation included the possibility of immunization and concerns for family safety.

**Conclusion:**

The findings indicated that nursing students are generally willing to assist as members of the healthcare team during disasters. However, the willingness to respond to infectious disease epidemics and man-made disasters was below the norm. Concerns about family health and the risk of disease transmission were identified as primary barriers. Addressing these concerns is crucial to enhance nursing students’ participation in disaster response.

**Supplementary Information:**

The online version contains supplementary material available at 10.1186/s12912-024-02088-4.

## Introduction

Formerly, it was believed that the healthcare sector’s performance was limited to the aftermath of disasters. However, the experiences of health organizations have revealed that a lack of proactive crisis preparedness often leads to significant inconsistencies during the post-disaster phase [1]. Research has indicated that societies with pre-disaster planning programs in place can markedly reduce the extent of damages caused by disasters [2].

The effectiveness and capability of treatment team members are pivotal components of disaster management, particularly in the provision of services during disasters or public health emergencies [3]. Experiences from different countries, including Australia, have underscored the challenges faced by healthcare and emergency medical service personnel and managers in responding to the escalating demand for such services, prompting the development of innovative response strategies. A key concern during disruptions is the ability to rapidly increase the number of personnel, especially within the nursing workforce [4]. As the largest contingent of treatment team members, nurses play a crucial role in providing direct care to victims in hospitals and responding to the needs of communities and individuals during disasters [3–5].

Nursing students represent a potential resource that could be leveraged to supplement the healthcare workforce during disasters [4]. Research indicates that nursing students possess the basis of knowledge, skills, and abilities that make them valuable and unique assets during disaster response efforts. Although they may lack the specialized training and certification required for clinical care, nursing students can perform various tasks that help alleviate the workload of experienced nurses in patient care. These tasks encompass both clinical services, such as wound management, vital sign monitoring, and triage, and non-clinical services, such as providing support to families [6].

Several studies have documented nursing students’ involvement of in disaster response efforts. For instance, following Hurricane Katrina in 2005 and 2008, nursing students from the University of Texas were involved in identifying and locating individuals who had been left behind. Similarly, between 2006 and 2008, nursing students in Indiana provided significant aid to victims of Hurricane Katrina [7]. These examples illustrate the important role that nursing students can play in disaster response, and highlight the value of their contributions in such situations.

A comparative study conducted by Dunlop et al. [8], reported the participation of nursing students in several disaster response efforts, including the Kentucky ice storms in 2009 and Hurricanes Gustav and Ike in 2008. In the aftermath of the 1995 Tokyo subway attack and the Hanshin-Awaji earthquake in Japan, nursing students collaborated with experienced nurses to provide assistance to victims. While national studies on this topic are limited, Rastegar et al. [9] described the effective involvement of volunteer student teams in responding to the 2012 Bam earthquake. However, studies have also indicated that both nurses and nursing students lack confidence in their ability to respond to disasters [10].

Healthcare workers’ willingness to respond to emergency situations is a critical component of the health system’s capacity to effectively manage disasters [5]. Factors such as the type of disaster, personal and familial concerns, and care responsibilities can impact the willingness of healthcare team members to participate in disaster response efforts [11]. Thus, understanding the factors that influence willingness to respond is crucial, regardless of successful examples of nursing students and other personnel in previous disasters. For instance, a study conducted in Saudi Arabia during the early stages of the Covid-19 pandemic found that only one-third of undergraduate medical science students and final-year medical students were willing to volunteer to assist [12]. Results of a study revealed that the overall desires of Canadian nurses to respond during an earthquake were higher than those of Israeli nurses [13].

Several studies have investigated the factors influencing nurses’ and other healthcare professionals’ willingness to respond to disasters. One such study by Adams and Berry found that concerns related to personal and family safety, caregiving responsibilities for dependent children, and the need to care for pets could diminish healthcare professionals’ willingness to respond to different types of disasters [14].

Given Iran’s unique geographical and geopolitical conditions, along with its significant vulnerability to natural and man-made disasters, understanding the willingness of healthcare team members to participate in disaster response efforts can provide valuable insights into capacity building and human resource management in the public health sector. However, limited information is available on the involvement of nursing students in disaster response in Iran, and no studies have been conducted with this specific focus. The importance of this issue is amplified during times of crisis such as the Covid-19 pandemic, which can lead to shortages and fatigue among healthcare personnel. To address this knowledge gap, this study was designed to investigate the factors influencing nursing students’ willingness of to participate in disaster response efforts.

## Method

This study employed a cross-sectional descriptive design, utilizing a census approach for data collection. Due to the absence of a standardized and appropriate instrument, a questionnaire was created using a deductive-inductive approach. The literature review informed the initial items, which were further expanded through a qualitative approach. Three open-ended questions were distributed to 12 nursing students using a structured self-expression questionnaire. The results were then analyzed using content analysis by two authors who are experts in conducting qualitative research, and included in the existing items. To ensure content validity, a 7-question scale judgment by an expert panel consisting of 12 nursing professionals, emergency medical professionals, and instrument development experts was employed. Face validity was assessed by 10 nursing students who participated in responding to a structured self-expression questionnaire, which helped identify ambiguous items. Reliability evaluation included test-retest, employing the intra-cluster correlation (ICC) for 37 nursing students, resulting in an acceptable ICC value of 0.874 [15]. Internal consistency, assessed using Cronbach’s alpha [16], demonstrated acceptable reliability (≥ 0.7) for all questionnaire sections. These students were excluded from the final research sample. Consequently, 243 out of 280 nursing students from Alborz University of Medical Sciences were included in the final study, and 234 of them completed the questionnaire. The response rate was 96.29%. Inclusion criteria encompassed being a nursing undergraduate student and expressing a willingness to participate.

The final questionnaire consisted of 55 questions categorized into three parts: Demographic Characteristics, General Questions, and Main Questions. General Questions gauged nursing students’ willingness to respond to various disasters and their moral obligations to participate. The main Questions explored the factors influencing career choice, disaster response activities, scenarios, facilitators, and barriers. The questionnaire was distributed to students through various online channels such as email, and social media including WhatsApp, and Telegram. To further increase the reach of the questionnaire, we enlisted student representatives in groups of students on social media. Both descriptive (mean and standard deviation) and inferential statistical analyses (one sample T-test, and Pearson correlation coefficient) were conducted on gathered data. The study findings, along with recommendations for their application, were disseminated to relevant stakeholders upon the completion of the study.

## Findings

The age range of the participants in this study was 17–40, with an average of 21.11 ± 3.16 years. Regarding other demographic factors, 219 participants (93.6%) were single, and 128 (54.7%) were female. Additionally, a large proportion of the participants (77.8%) were currently unemployed and 24.4% of the students resided in the dormitory. A total of 13.2% of participants reported having volunteered during critical situations in the past. The demographic characteristics are shown in Table 1.

**Table 1:** Demographic characteristics of participants (*n* = 234)

Variable		No	percentage
**Sex**	male	106	45.3
	female	128	54.7
**Marital status**	single	219	93.6
	married	15	6.4
**Living in dormitory**	yes	57	24.4
	no	177	75.6
**Occupational status**	Only student	182	77.8
	Working in the field of medical sciences	25	10.7
	Working in a field not related to medical sciences	27	11.5
**Number of kids**	0	225	96.2
	1	5	2.1
	2	4	1.7
**Semester**	4≥	168	71.8
	4<	66	28.2
**Age**	mean ± SD	3.16 ± 21.11	
	Min- Max	17–40	

The study’s findings indicated that a significant majority of nursing students (86.8%) perceived disaster management themes as crucial to the nursing discipline. Moreover, according to the same proportion of participants, the university did not encourage them to join groups offering disaster aid. Of note, 39.2% of the participants who participated in the approved aid and relief classes did so independently.

Table 2 displays the nursing students’ mean willingness scores for disaster response, both “in general” and for “specific disaster types”. The findings revealed that students’ willingness to respond to man-made disasters and infectious epidemics fell below the average norm of willingness (average = 3). However, their desire to respond to natural disasters was noteworthy, with a mean score of 3.32, indicating a significant and desirable level of willingness.

**Table 2:** One-sample t-test to compare the average willingness of nursing students to respond in disasters

Variable	mean	standard deviation	t	*p*
**Willingness to respond to disasters in general**	3.32	0.83	5.98	> 0.001
**Willingness to respond to disasters in natural disasters**	3.15	1.18	2.04	0.042
**Willingness to respond to man-made disasters**	2.60	1.35	4.49	> 0.001
**Responding to infectious epidemics**	2.94	1.26	0.61	0.537

The results indicated that among the reported disaster scenarios, “an earthquake” had the highest average willingness score of 3.69, whereas “cholera pandemic” had the lowest score of 3.12. Notably, the students’ willingness to participate in all scenarios exceeded the standard average score of three, signifying their readiness to engage in various disaster response situations.

Table 3 presents nursing students’ willingness levels for various disaster response activities. The findings revealed that “bedside caring (triage and treatment)” had the highest average willingness score of 4.05, while “participating in personal hygiene of patients” received the lowest score of 2.17. One sample T-test was used to compare the average score of students’ willingness to respond to disasters in various activities. Generally, students’ willingness to engage in all reported disaster response activities was higher than the average standard, except for activities such as patient personal hygiene, caring for hospital employees’ children, assisting with the management of corpses, and documenting and reporting.

**Table 3:** Descriptive indicators of students’ willingness to respond to disasters in various activities (One sample T-test)

Activity	Very little (%)	Little (%)	Average (%)	High (%)	Very high (%)	Mean (± SD)	Value
**Participating in feeding patients**	7.7	12.8	35.9	31.6	12	3.27(1.07)	*P* < 0.001 t = 3.89
**Participating in the personal hygiene of patients**	33.3	29.1	27	7.3	3	2.17(1.06)	*P* < 0.001 t = 11.81
**Doing administrative and non-care or non-clinical work**	10.7	18.4	30.8	24.8	15.4	3.15(1.20)	*p* < 0.001 t = 2.01
**Caring for children of hospital employees**	15.8	25.2	31.6	16.2	11.1	2.81(1.20)	*P* = 0.001 t = 2.32
**Performing basic clinical care (such as vital signs)**	3.8	5.1	31.2	32.9	26.9	3.73(1.03)	*p* < 0.001 t = 10.93
**Bedside caring (triage and treatment)**	1.7	3.8	17.1	42.3	35	4.05(0.91)	*p* < 0.001 t = 17.56
**Psychological support for patients and their families**	3.8	8.1	29.5	34.6	23.9	3.66(1.04)	*p* < 0.001 t = 9.72
**Patient education and patient follow-up**	5.1	5.6	30.8	34.6	23.9	3.66(1.06)	*p* < 0.001 t = 9.61
**Helping in collecting public donations**	9.8	20.5	32.9	21.4	15.4	3.11(1.19)	*P* = 0.126 t = 1.53
**Assisting in the management of corpses**	28.2	26.1	26.1	10.7	9	2.46(1.25)	*p* < 0.001 t=-6.57
**Assisting in the public health issues, and environmental concerns resulting from the crisis**	6	13.7	37.2	30.3	12.8	3.30(1.05)	*p* < 0.001 t=-4.41
**Participating in the rescue process**	4.3	9.4	22.2	38	26.1	3.72(1.08)	*p* < 0.001 t = 10.2
**Documenting and Reporting**	15.4	23.1	29.1	23.1	9.4	2.88(1.20)	*P* = 0.129 t = 1.52

Table 4 displays the mean score for nursing students’ perceived responsibility and moral obligation to assist individuals in need (3.90). Notably, this score was higher than the average level of willingness (average = 3), indicating that the students felt a strong sense of duty to help those in need during disaster situations.

**Table 4:** The degree of moral obligation and personal belief to respond to disasters in nursing students (One-sample T-test)

Degree of willingness						Mean (± SD)
Very little N (%)	Little N (%)	Average N (%)	High N (%)	Very high N (%)	At all N (%)	
9 (3.8)	24 (10.3)	53 (22.6)	43 (18.4)	105 (44.9)		3.90 (1.19)

*p* < 0.001, t = 11.54

The findings revealed that the most significant facilitator factor for nursing students’ willingness to engage in disaster response was the “possibility of vaccination and prevention for the family,” with a mean score of 4.20. By contrast, the occurrence of a disaster in the location or city of residence, particularly with ethnic or racial affiliation, was perceived as the least facilitator factor, with a mean score of 3.66. Moreover, the most significant barrier to students’ willingness to participate was their “concern for family health and safety,” with a mean score of 4.09. Conversely, “obstructing parents to participate and respond during disasters” was the least significant barrier, with a mean score of 3.31.

This study identified a significant relationship between nursing students’ academic semesters and their disaster response capacity (*P* = 0.05). Specifically, fourth-semester students exhibited a higher average willingness to respond, whereas the willingness of other nursing students was lower (*P* = 0.013). Additionally, no significant differences were observed in nursing students’ willingness to respond based on demographic factors, except for prior volunteer experience (*P* > 0.05).

Table 5 presents the results of Pearson’s correlation coefficient analysis, which indicated a positive and statistically significant relationship between all factors influencing nursing field choice (excluding entrance exam scores) and nursing students’ willingness to respond to disasters (*p* < 0.01). Notably, the factor with the highest correlation was “serving the people.”

**Table 5:** Pearson correlation coefficient between research variables (*n* = 234)

Research variables	1	2	3	4	5	6	7
**Income**	1						
**Serving**	0.159^b^	1					
**Effective role**	0.164^b^	0.613^a^	1				
**Job position**	0.212^a^	0.492^a^	0.618^a^	1			
**Continuing education**	0.192 ^a^	0.403^a^	0.485 ^a^	0.674^a^	1		
**Disciplinary tendencies**	0.203^a^	0.214^a^	0.232 ^a^	0.399^a^	0.560^a^	1	
**entrance exam rank**	0.121	0.085	0.116	0.126	0.07	0.047	1
**Willingness level**	0.204 ^a^	0.514^a^	0.492^a^	0.387^a^	0.360^a^	0.219 a	0.025

a(*p* < 0.01) b (*p* < 0.05)

## Discussion

The vital role of health care providers in health systems is undeniable. In times of disasters and public health emergencies, the importance of this valuable resource for organizations multiplies. However, shortage of human resources has always been a challenge in crisis management. Nurses play a significant role in disaster management as they are among the largest healthcare providers. Under normal conditions, Iran is facing a shortage of nurses. Distribution of nurses in Iran is generally lower than the average of other countries, and this ratio is lower in some wards and higher than the average in others [17]. The shortage of nursing staff was exacerbated by the Covid-19 pandemic for various reasons, including dealing with a new disease, being away from home, working long hours, and confronting moral dilemmas [18]. Meanwhile, a study showed that the readiness of hospitals in Iran to address disasters is not at its optimal level. Moreover, the effective and efficient management of resources is considered one of the foremost challenges [19]. Iran is one of the ten most vulnerable countries in the world, and almost 90% of its population is exposed to natural disasters. Considering this situation, it is imperative to prioritize disaster preparedness [20]. Alternative personnel, such as nursing students have been suggested to manage the shortage of nursing staff during disasters. In this regard, some countries, including Spain and England, although restricted the presence of nursing students in clinical settings, final-year volunteer students were allowed to attend hospitals and help the nursing team [21]. Furthermore, in previous infectious pandemics, such as SARS and MERS, volunteer nursing students were present [22].

Although nursing students have a strong desire to serve during health crises, their employment has been questioned in several studies due to their lack of sufficient experience [22, 23]. According to the findings of the present study, students’ general tendency to participate in and respond to disasters was higher than the average level of willingness. However, the willingness is mainly related to natural disasters, except for infectious events. The willingness of healthcare providers to assist during disasters usually varies based on factors such as the type of disaster, personal and familial obligations, and caregiving responsibilities. Choi’s [11] study revealed that, in contrast to natural calamities and environmental and weather-related disasters, healthcare providers are less willing to respond to “dirty disasters’’, such as pandemics, chemical attacks, bioterrorism, and bombings.

Consistent with the current study, previous research has suggested that nursing students are more willing to respond to natural disasters than to man-made disasters. Specifically, Blackwood’s [7] study found that 92.7% of nursing students were willing to respond to natural disasters compared to 80% for man-made disasters. This trend was also observed among medical students, as reported in Choi’s [11] study. However, a study conducted in Saudi Arabia revealed that only one-third of medical science undergraduates and final-year medical students expressed a willingness to volunteer for the Covid-19 pandemic [12].

Similar findings in the other studies, indicating that nursing students had little desire to participate in the Covid-19 pandemic and considered it an obstacle to participation [24, 25]. Given the phenomenon of globalization and changing patterns of disease spread across different regions, it is imperative to conduct a comprehensive evaluation of the nursing curricula, considering emerging and re-emerging diseases [26].

One noteworthy finding of the current study is that over 50% of the students expressed a strong moral commitment and personal belief in assisting those in need during disasters. In line with nursing professional ethics guidelines, it is essential to institutionalize empathy and accountability as ethical dimensions in the professional activity of students. This finding is in line with the findings of other studies [18, 21]. The findings of the other study also demonstrated a significant and positive relationship between nurses’ professional ethics and responsiveness [27]. A study conducted in Saudi Arabia found that students who did not perceive participation in disaster relief activities as a moral obligation were less inclined to engage in such activities [12].

The current study identified immunization opportunities and the prevention of family infections as the most significant facilitators, while concerns for family safety were the most significant barriers. Notably, the study’s findings revealed that students’ families were both the most significant facilitator and the most significant barrier, particularly during infectious epidemics like the Covid-19 pandemic. Remarkably, the results of this study were influenced by the timing and context of the pandemic. In such situations, healthcare personnel, particularly volunteers, are primarily concerned about the potential transmission of the disease from the medical environment to their home environment. This finding has also been reported in other studies. A qualitative systematic review study demonstrated that concern for family was one of the primary themes. This concern was especially prominent regarding the transmission of the pathogen to parents and children [28]. The finding of a study by Delgado et al. [21], revealed that students’ biggest concern was the potential of infecting their families. As a result, many individuals chose to stay in the dormitory and modify their communication methods and interactions with their families and others. Among the significant facilitators of willingness to participate in disasters, this study identified comfort facilities and the availability of personal protective equipment for volunteer personnel. This finding was consistent with Delgado’s study [21]. However, caring for children and pets as barriers to participating in disasters identified in the other study [11].

One of the primary concerns is the perceived incapability of students to effectively response facing disasters due to inadequate training, and lack of preparedness in addressing and managing such catastrophic circumstances [7, 29].

The lack of specific competencies, including knowledge and related skills, among nurses has been identified as a major barrier to an effective emergency response. These competencies are considered the foundation for training courses aimed at improving organizational readiness and response capabilities. Nursing students express a willingness to assist in disaster response, but may lack confidence in their ability to do so [30]. Nevertheless, supposedly despite their lack of expertise, nursing students experience lower levels of stress and are highly motivated to participate in disaster response because of their strong self-confidence [31]. As a result, the curricula for all Iranian medical science students have been updated to include two credits for disaster risk management, reflecting the recognition of the importance of education in promoting the preparedness and motivation of medical science students.

Moreover, this study indicated that activities such as triage and treatment, clinical care, and rescue were more desirable than other activities among nursing students. Although it may seem that specialized groups prefer to engage in activities that align with their specific roles and tasks, the findings present conflicting evidence. For instance, a cross-sectional study by Yonge et al. [32], found that 60–70% of the nursing students reported working in various roles within hospitals, such as assisting with patient feeding (67.2%), performing administrative tasks (60.8%), providing food and snacks for staff (64.8%), or performing tasks as needed (68.9%). Similarly, a similar proportion of students (65.1%) expressed willingness to volunteer for tasks such as answering phones, caring for neighbors (69.5%), or purchasing groceries for patients (65.2%).

The qualitative responses from this study included three respondents who expressed concerns that nursing students were not sufficiently prepared to serve as volunteer nurses. Conversely, two respondents felt that performing administrative and clerical tasks or engaging in non-specialized volunteer work was not an effective use of their skills.

Although a previous research highlighted the importance of demographic factors in determining disaster response [32], the present study found that only academic semesters were significantly associated with willingness to respond as the fourth-semester nursing students were more willing to respond to disasters than their peers. this finding is not in line with the findings of other study [12]. In the other investigation which conducted in Israel, results revealed that women under 40 and nurses were more likely to be absent than other subgroups [13]. It should be noted that a significant proportion of the study participants were unmarried women. It is conceivable that married women may have more responsibilities such as childcare, which could limit their willingness to respond to disasters.

Examining the correlation between nursing students’ choice of nursing field and their willingness to respond to disasters showed that the factors of serving and having an effective role in society were significantly related to the willingness to respond. Similarly, a previous study found that the desire to serve others, religious beliefs, and charitable motivations were strong motivators for volunteers, along with patriotism and gaining practical experience [12].

Furthermore, the study found that nursing students with a history of volunteering demonstrated a higher average willingness to assist in disaster relief than their peers. This finding is consistent with the results of a previous study which also found that professional experience with disaster teams was associated with a greater willingness to assist in disaster relief [11].

Understanding the level of willingness of healthcare providers to participate in disaster response can aid in disaster management and inform manpower planning strategies for health organizations and public health officials. To improve willingness, it is important to consider factors such as ensuring a safe environment, providing sufficient protective equipment, distributing available resources effectively, and increasing the number of personnel who can take on the responsibilities of nurses in emergency departments.

## Study limitations

One limitation was the low participation rate among nursing students, which was addressed through email distribution and reminders. This study acknowledges this limitation and emphasizes the advantage of having a student distributor, which may have enhanced attention and participation. Another limitation of this study was the use of convenience sampling from a single setting, which may limit the generalizability of the result.

## Conclusion

The study findings suggest that nursing students are generally willing to assist as members of the healthcare team during natural disasters but exhibit less willingness during pandemics and man-made disasters. Concerns about family health and the risk of disease transmission to loved ones were identified as primary barriers to nursing students’ participation and accountability. It is crucial to develop comprehensive disaster preparedness plans that incorporate a range of educational techniques to effectively recruit nursing students as alternative staff members during human resource disasters, particularly in nursing. The emotional characteristics of Eastern families, especially in Iran, and their unique relationships reveal the necessity of designing a family disaster plan with the aim of providing effective human resources during disasters. Furthermore, addressing students’ concerns, particularly those related to man-made and infectious disasters, will require appropriate personal protective equipment and vaccines when needed.

### Electronic supplementary material

Below is the link to the electronic supplementary material.

Supplementary Material 1

## Data Availability

Data is provided within the manuscript files.
