# Supplementary material for: Nursing students’ willingness to respond in disasters: a cross sectional study of facilitators and barriers
Source: BMC Nurs. 2024 Jun 20;23:416. doi: 10.1186/s12912-024-02088-4 (PMC11188220; doi:10.1186/s12912-024-02088-4)
Supplement: Supplementary file 1 — Supplementary Material 1 [file 12912_2024_2088_MOESM1_ESM.docx]

**Dear student**

The purpose of this questionnaire is to investigate nursing students' willingness to participate in disaster response and the factors that influence it. Your accurate responses will assist the research team in collecting precise data, leading to accurate findings and ultimately, effective implications.

**Demographic characteristics:**

Age: Gender: Marital Status: Semester:

Living in a dormitory: Employment status: Number of kids:

Are you currently a volunteer member in a disaster response organization such as the Red Crescent?

Do you have the experience of being a volunteer force in a crisis situation?

**General questions**

1. How much are you willing to participate and respond to a natural disaster such as an earthquake, flood, etc.?

| At all  1 | Very little  2 | little  3 | So-so  4 | much  5 | Very much  6 |
| --- | --- | --- | --- | --- | --- |
|  |  |  |  |  |  |

1. How much are you willing to participate and respond to a man-made disaster such as a terrorist attack, release of dangerous substances, fire, etc.?

| At all | Very little | little | So-So | much | Very much |
| --- | --- | --- | --- | --- | --- |
|  |  |  |  |  |  |

1. How much are you willing to participate and respond in a disaster that leads to a pandemic and its origin is not clear, such as influenza, Covid, etc.?

| At all | Very little | little | So-So | much | Very much |
| --- | --- | --- | --- | --- | --- |
|  |  |  |  |  |  |

1. In times of disaster, nursing students are morally obligated to help others.

| strongly disagree | Disagree | Neutral | Agree | strongly agree |
| --- | --- | --- | --- | --- |
|  |  |  |  |  |

**Main questions**

**How much did each of the following factors influence your choice of nursing field?**

|  | **Factors** | Very little | little | So-So | much | Very much |
| --- | --- | --- | --- | --- | --- | --- |
| 1 | The possibility of employment and earning in nursing is high. |  |  |  |  |  |
| 2 | It is important for me to be a member of a team that saves people. |  |  |  |  |  |
| 3 | With nursing science, I can be an effective person in the family and society. |  |  |  |  |  |
| 4 | Different job positions in this field are attractive to me. |  |  |  |  |  |
| 5 | Continuing education from nursing to other fields is possible. |  |  |  |  |  |
| 6 | Entrance exam rank |  |  |  |  |  |
|  | **etc.** | | | | | |

**How willing are you to do the following activities when disasters occur?**

|  | **Activity** | Very little | little | So-So | much | | Very much |
| --- | --- | --- | --- | --- | --- | --- | --- |
| 1 | Participating in feeding patients |  |  |  |  |  | |
| 2 | Participating in personal hygiene of patients |  |  |  |  |  | |
| 3 | Doing administrative tasks |  |  |  |  |  | |
| 4 | Caring for children of hospital employees |  |  |  |  |  | |
| 5 | Performing basic clinical care (such as vital signs) |  |  |  |  |  | |
| 6 | Bedside caring (triage and treatment) |  |  |  |  |  | |
| 7 | Psychological support for patients and their families |  |  |  |  |  | |
| 8 | Patient education and patient follow-up after discharge |  |  |  |  |  | |
| 9 | Helping in collecting public donations |  |  |  |  |  | |
| 10 | Assisting in the management of corpses |  |  |  |  |  | |
| 11 | Assisting in the public health issues, and environmental concerns resulting from the crisis |  |  |  |  |  | |
| 12 | Participating in the rescue process |  |  |  |  |  | |
| 13 | Documenting and reporting |  |  |  |  |  | |
|  | etc. | | | | | | |

**Considering the following scenarios, determine how much you are willing to participate to each of the following incidents:**

|  | **Scenario** | Very little | little | So-So | much | Very much |
| --- | --- | --- | --- | --- | --- | --- |
| 1 | Earthquake: An earthquake with a magnitude of 7 shook the city and caused serious damage to roads and buildings, electricity was cut off and many people were injured, missing and killed. |  |  |  |  |  |
| 2 | Heavy snowfall: Where you live, there was a winter storm with 15 cm of snow and 8 cm of ice, and many people were injured. |  |  |  |  |  |
| 3 | Dust storm and fine dust: A severe storm has damaged hundreds of people in the city and a large number of injured have been taken to the hospital. |  |  |  |  |  |
| 4 | Flood: Heavy rain has caused significant flooding in the city and hundreds of people have become homeless. |  |  |  |  |  |
| 5 | Refinery explosion: 200 people were seriously injured and sent to the hospital. |  |  |  |  |  |
| 6 | Infectious disease epidemic: 600 reported cases of cholera, hospitals are rapidly filling capacity. |  |  |  |  |  |
| 7 | Pandemic of infectious diseases: the covid-19 pandemic has been confirmed by the World Health Organization and the capacity of hospitals has been completed. |  |  |  |  |  |
| 8 | Terrorist incident: A bomb exploded in a religious center and hundreds of people were injured. A terrorist group has claimed responsibility for the explosion. |  |  |  |  |  |
| 9 | Fire: An old commercial tower caught fire and hundreds of residents and visitors were trapped and a significant number of injured are expected to be sent to the hospital. |  |  |  |  |  |
| 10 | Water pollution: An unknown source caused urban water pollution and hundreds of people with symptoms of gastrointestinal poisoning went to medical centers. |  |  |  |  |  |

**To what extent does each of the following factors facilitate your willingness to participate in disasters?**

|  | **Facilitating factors** | Very little | little | So-So | much | Very much |
| --- | --- | --- | --- | --- | --- | --- |
| 1 | The presence of protective equipment and vaccines if needed |  |  |  |  |  |
| 2 | The possibility of vaccination and prevention for the family |  |  |  |  |  |
| 3 | The possibility of accommodation in a dormitory or temporary residence |  |  |  |  |  |
| 4 | The presence of suitable amenities during service |  |  |  |  |  |
| 5 | Passing disaster preparedness courses before disaster strikes |  |  |  |  |  |
| 6 | Gain work experience for the future and improve clinical skills |  |  |  |  |  |
| 7 | Accompanying and attending friends and classmates in service centers |  |  |  |  |  |
| 8 | Receive allowance in exchange for the service provided |  |  |  |  |  |
| 9 | The centers have a serious need for auxiliary forces |  |  |  |  |  |
| 10 | The occurrence of a disaster in the place or city of residence (ethnic or racial affiliation) |  |  |  |  |  |
| 11 | Granting points and job promotion in the future (equalization with academic units - employment priority, points in continuing education, etc.) |  |  |  |  |  |
| 12 | Payment of insurance and damages in case of death or physical and mental injury |  |  |  |  |  |
|  | etc. | | | | | |

**To what extent do each of the following factors hinder your participation during disasters?**

|  | **barriers** | | Very little | little | | So-So | much | Very much |
| --- | --- | --- | --- | --- | --- | --- | --- | --- |
| 1 | Fear about your personal safety and health | |  |  | |  |  |  |
| 2 | Fear about the health and safety of the family | |  |  | |  |  |  |
| 3 | Responsibility for taking care of people in the family | |  |  | |  |  |  |
| 4 | Type of disaster (causing cause such as floods, earthquakes, pandemics, etc.) | |  |  | |  |  |  |
| 5 | Preventing parents from participating and responding during disasters | |  |  | |  |  |  |
| 6 | Overwhelming pressure and heavy workload | |  |  | |  |  |  |
| 7 | Imbalance between hard work and income |  | | |  |  |  |  |
| 8 | The distance between the service centers and the place of residence |  | | |  |  |  |  |
| 9 | Unclear role and confusion in the crisis team |  | | |  |  |  |  |
| 10 | The impossibility of predicting the course of the crisis (the duration of the disaster and possible complications) |  | | |  |  |  |  |
| 11 | Disruption in the study process at the university (attending classes and related exams) |  | | |  |  |  |  |
| 12 | Disruption in the current employment situation (due to being in a crisis) |  | | |  |  |  |  |
| 13 | The impossibility of predicting the course of the crisis (the duration of the disaster and possible complications) |  | | |  |  |  |  |
| 14 | Disruption in the study process at the university (attending classes and related exams) |  | | |  |  |  |  |
| 15 | Disruption in the current employment situation (due to being in a crisis) |  | | |  |  |  |  |
|  | etc. | | | | | | | |

**Is there anything else you would like to say?**
